# Rocky Mountain Spotted Fever in Mexico: A Call to Action

**DOI:** 10.4269/ajtmh.24-0265

**Published:** 2024-09-17

**Authors:** Gerardo Álvarez-Hernández, Ruy López-Ridaura, Ricardo Cortés-Alcalá, Gabriel García Rodríguez, J. R. Tadeo Calleja-López, Cristian N. Rivera-Rosas, José Luis Alomía-Zegarra, Maureen Brophy, Carina Berenice Brito-Lorán, Maria del Carmen Candia-Plata, Santa Elizabeth Ceballos-Liceaga, Fabián Correa-Morales, Karla R. Dzul-Rosado, Janet Foley, José Manuel Galván-Moroyoqui, Roman Ganta, Verónica Gutiérrez-Cedillo, Néstor Saúl Hernández-Milán, Andrés M. López-Pérez, Luis Fernando López-Soto, Juan Manuel Martínez-Soto, Ana Lourdes Mata-Pineda, Christopher D. Paddock, Irma Leticia J. Ruiz-González, Juan Edmundo Salinas-Aguirre, Johanna S. Salzer, Sokani Sánchez-Montes, Adriana Soto-Guzmán, Óscar Tamez-Rivera, David M. Wagner, David H. Walker

**Affiliations:** ^1^ Departamento de Medicina y Ciencias de la Salud, Universidad de Sonora, Hermosillo, Mexico;; ^2^ Subsecretaría de Prevención y Promoción de la Salud, Secretaría de Salud, Ciudad de México, Mexico;; ^3^ Centro Nacional de Programas Preventivos y Control de Enfermedades, Secretaría de Salud, Ciudad de México, Mexico;; ^4^ Dirección General de Epidemiología, Secretaría de Salud, Ciudad de México, Mexico;; ^5^ Secretaría de Salud Pública del Estado de Sonora, Hermosillo, Mexico;; ^6^ National Center for Emerging and Zoonotic Infectious Diseases, Centers for Disease Control and Prevention, Atlanta, Georgia;; ^7^ Instituto Nacional de Diagnóstico y Referencia Epidemiológicos (InDRE) “Dr. Manuel Martínez Báez,” Secretaría de Salud, Ciudad de México, Mexico;; ^8^ Centro de Investigaciones Regionales “Dr. Hideyo Noguchi,” Universidad Autónoma de Yucatán, Mérida, Mexico;; ^9^ Department of Medicine and Epidemiology, School of Veterinary Medicine, University of California, Davis, California;; ^10^ Department of Veterinary Pathobiology, College of Veterinary Medicine, University of Missouri, Columbia, Missouri;; ^11^ Secretaría de Salud del Estado de Baja California, Mexicali, Mexico;; ^12^ Red de Biología y Conservación de Vertebrados, Instituto de Ecología, Xalapa, Mexico;; ^13^ Secretaría de Salud del Estado de Chihuahua, Chihuahua, Mexico;; ^14^ Secretaría de Salud del Estado de Coahuila, Saltillo, Mexico;; ^15^ Facultad de Ciencias Biológicas y Agrícolas, Región Poza Rica-Tuxpan, Universidad Veracruzana, Tuxpan de Rodríguez Cano, Mexico;; ^16^ Tecnológico de Monterrey, Escuela de Medicina y Ciencias de la Salud, Monterrey, Mexico;; ^17^ Northern Arizona University, Flagstaff, Arizona;; ^18^ Department of Pathology, University of Texas Medical Branch, Galveston, Texas

## Abstract

Rocky Mountain spotted fever (RMSF) is an ongoing public health crisis in Mexico, particularly in states bordering the United States. The national highest incidence and mortality of RMSF occur in this region, resulting in a case-fatality rate that ranges annually between 10% and 50%, primarily affecting vulnerable groups such as children, elderly adults, and persons living in poverty. Multiple biological, environmental, and social determinants can explain its growing presence throughout the country and how it challenges the health system and society. It is necessary to integrate resources and capacities from health authorities, research centers, and society to succeed in dealing with this problem. Through a scientific symposium, a group of academicians, U.S. health officials, and Mexican health authorities met on November 8–10, 2023, in Hermosillo, Mexico, to discuss the current situation of RMSF across the country and the challenges associated with its occurrence. An urgent call for action to improve national capacity against RMSF in the aspects of epidemiological and acarological surveillance, diagnosis, medical care, case and outbreak prevention, health promotion, and research was urged by the experts. The One Health approach is a proven multidisciplinary strategy to integrate policies and interventions to mitigate and prevent the burden of cases, deaths, and suffering caused by RMSF in Mexico.

## INTRODUCTION

Rocky Mountain spotted fever (RMSF) is a severe, tickborne zoonotic disease caused by *Rickettsia rickettsii.* The pathogen can be transmitted to human beings through the bite of infected hard ticks of the genera *Rhipicephalus*, *Dermacentor*, *Amblyomma*, and *Haemaphysalis*.^1^^,^^2^ The disease has remained a public health threat affecting inhabitants of several countries across the Americas.^3^^–^^5^ Nowhere is this more evident than in northern Mexico, primarily along the United States-Mexican border, where extremely high rates of morbidity and mortality disproportionately impact vulnerable populations such as children, older adults, migrants, and people living in poverty.^6^^–^^9^

With that in mind, the Department of Medicine and Health Sciences of the University of Sonora, Mexico, convened international and national experts, as well as U.S. health officials and Mexican health authorities, to discuss the epidemiological situation and the challenges to be addressed. This was accomplished through a symposium held from November 8–10, 2023, in Hermosillo, Mexico. The symposium was attended by 171 health care workers and academicians and 230 medical students. The scientific program included 20 speakers actively affiliated with 17 different health institutions and U.S. and Mexican governmental agencies and universities. The meeting’s schedule was structured around 22 presentations organized into three main topics: 1) epidemiology and public health, 2) the One Health approach, and 3) research insights. The key messages and recommendations are summarized in the following sections.

## EPIDEMIOLOGICAL PROFILE

Rocky Mountain spotted fever is an ongoing public health problem in Mexico, with hyperendemic foci in different localities along the U.S.-Mexico border, where cases and deaths have been registered since the early 2000s, when it reemerged after decades of apparent rarity.^6^^,^^10^^–^^13^ Since then, scientific reports have also recorded human cases in Yucatan,^14^^–^^16^ Sinaloa,^17^ Jalisco,^18^ and Chiapas.^19^ Additionally, cases from other states across the country except Tlaxcala have been consistently notified through the National System of Epidemiological Surveillance.^20^

**Figure 1.**
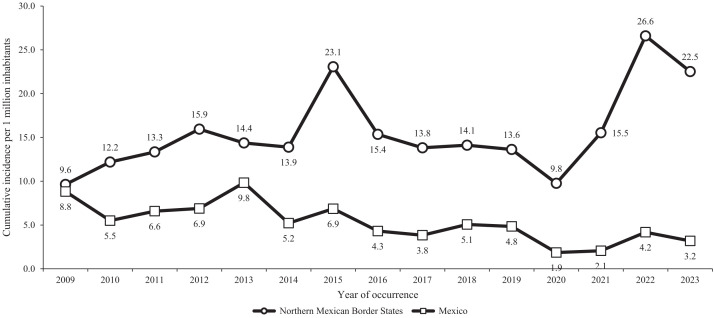
Comparison of human incidence of Rocky Mountain spotted fever and spotted fever group *Rickettsia* in Mexican states bordering the United States (including data from Baja California, Sonora, Chihuahua, Coahuila, and Nuevo Leon) and in Mexico as a country, 2009–2023. Denominators for population estimates were obtained from the National Population Council of Mexico (population projections 2000–2023).

During the 2009–2023 period, at the national level, there were 9,153 reported cases of spotted fever rickettsioses (SFR),^20^ which includes RMSF and the spotted fever group of *Rickettsia*, with a decrease in incidence throughout the period, going from 8.8 cases per million inhabitants in 2009 to 3.2 cases per million inhabitants in 2023. Despite this, an increase in morbidity was observed in the northern region of the country during the same period, where 4,373 cases of SFR were reported in five of the six Mexican states bordering the United States (Figure 1). In this region, hyperendemic foci of the disease have been documented in Hermosillo,^21^^,^^22^ Mexicali,^7^ Saltillo,^8^^,^^13^ Monterrey,^9^ and Tijuana.^23^ Additionally, in the same region, 1,345 deaths were identified, resulting in a case-fatality rate (CFR) of 30.8%, with Sonora presenting the highest (541/1,349 = 37.9%) and Nuevo Leon the lowest (88/414 = 21.3%) CFRs (Table 1). This fatal pattern resembles the epidemiological profile seen in the United States during the preantibiotic era,^24^^,^^25^ reinforcing the seriousness of the current situation. It was agreed that numerous factors could explain these devastating CFRs, including regional circulation of highly virulent *Rickettsia* strains,^26^ emergent environmental phenomena, high tick load, high numbers of free-roaming dogs, high rates of recruitment of puppies lacking immunological exposure, low herd immunity, and limitations in the response capacity.

**Table 1:** Registered cases, deaths, and case-fatality rates of Rocky Mountain spotted fever and spotted fever group *Rickettsia* in selected states of the northern border of Mexico and the northern region by year of occurrence, 2009–2023

Year	Baja California*		Coahuila^†^		Chihuahua^‡^		Nuevo Leon^§^		Sonora^‖^		Northern Region		
	Cases	Deaths	Cases	Deaths	Cases	Deaths	Cases	Deaths	Cases	Deaths	Cases	Deaths	CFR (%)
2009	5	5	0	0	0	0	0	0	156	15	161	20	12.4
2010	117	8	0	0	0	0	0	0	90	16	207	24	11.6
2011	109	6	0	0	0	0	0	0	121	17	230	23	10.0
2012	96	9	62	9	6	0	0	0	115	19	279	37	13.3
2013	90	23	48	13	2	1	30	0	85	21	255	58	22.7
2014	80	30	16	7	6	3	47	1	101	26	250	67	26.8
2015	122	34	17	1	28	9	75	0	179	86	421	130	30.9
2016	62	21	14	2	55	17	29	0	124	48	284	88	31.0
2017	49	22	6	3	78	31	24	0	102	43	259	99	38.2
2018	40	12	10	6	77	28	36	0	105	46	268	92	34.3
2019	27	10	21	4	99	31	29	0	86	33	262	78	29.8
2020	25	10	10	5	74	32	15	0	66	20	190	67	35.3
2021	46	17	10	6	89	30	3	0	158	42	306	95	31.0
2022	90	37	29	21	166	49	55	35	190	92	530	234	44.2
2023	93	39	34	20	155	57	71	52	118	65	471	233	49.5
Total	1,051	283	277	97	835	288	414	88	1,429	541	4,373	1,345	–
CFR (%)	26.90		35.01		34.50		21.30		37.90		30.76		

CFR = case-fatality rate.

* These data are from the Public Health Services Institute, Baja California, Mexico. Epidemiological surveillance (special system, rickettsiosis). Data not publicly available.

^†^ These data are from the Ministry of Health, Chihuahua, Mexico. Epidemiological surveillance (special system, rickettsiosis). Data not publicly available.

^‡^ These data are from the Ministry of Health, Coahuila, Mexico. Special epidemiological surveillance system, rickettsiosis. Data not publicly available.

^§^ These data are from the Government of Mexico.^67^

^‖^ These data are from the Government of Sonora.^68^

An observation shared by the speakers is that the majority of confirmed cases of RMSF in Mexico involved children and adolescents, with almost half of the incidence and mortality occurring in this group.^9^^,^^11^^,^^13^^,^^27^ For instance, in Sonora during the period 2009–2023, 51.2% (724) of confirmed cases and 28.3% (153) of 541 registered deaths affected the pediatric segment.^28^^,^^29^ Additionally, the speakers highlighted that almost two-thirds of cases and deaths occurred in patients without medical insurance. In the north of Mexico, the epidemiological profile of RMSF accentuates its daunting impact on vulnerable individuals and socially disadvantaged populations. Therefore, the population pattern of the disease should be carefully considered in the planning and implementation of medical and sanitary interventions across Mexico, prioritizing efforts and resources in regions of high endemicity.^6^^,^^7^^,^^9^

Many conference speakers signaled a need to improve the national, state, and local epidemiological surveillance systems to strengthen the quality of statistics about the magnitude and impact of RMSF. Discrepancies and delays in published data are frequent in official sources of information, prompting inaccuracies in estimating the true morbidity and mortality of the disease across the country. Such discrepancies can cause inaccuracies in characterizing the epidemiological profile and an underestimation of the deleterious effect of RMSF and other rickettsial diseases at the population level.^6^

Emphasis was placed on the importance of strengthening the infrastructure, techniques, and capability of regional laboratories to confirm cases and outbreaks of the disease and to differentiate it from other endemic diseases that mimic its clinical picture, mainly dengue and COVID-19.^30^^,^^31^ In this context, national advances in laboratory capacity were underscored. For instance, there exists a national network comprised of 15 state laboratories dedicated to confirming rickettsioses using either polymerase chain reaction (PCR) or immunofluorescence indirect serologic assay. During the period 2018–2023, this national network was able to identify either the presence of *R. rickettsii* in biological samples or antibodies against the pathogen. In addition, other research groups have supported regional efforts to confirm cases and outbreaks. Nevertheless, the need for innovative methods to improve the promptness and accuracy of the current molecular and serological techniques was deliberated. The lateral-flow assay and other techniques from the genomic and proteomic approaches were introduced as novel alternatives,^32^^,^^33^ potentially serving in the near future as useful point-of-care tools to confirm RMSF cases, particularly in vulnerable communities with difficult access to health care.

Specific areas of interest considered by the speakers included the discussion of historical aspects of RMSF, the role played by social determinants, and communication as a fundamental strategy for its prevention and control. In addition, emphasis was placed on the need to strengthen educational interventions for health care workers (HCW) and the community, with the goal to improve prompt diagnosis and timely treatment of cases and outbreaks. These recommendations have been consistently advocated by expert panels.^31^^,^^34^ In this regard, there was a remarkable call to reactivate the national production of doxycycline to ensure its timely access, in both pediatric and intravenous formulations, for the care of children and severe cases in regions of endemicity in Mexico.

Finally, the experts concluded that governmental agencies should allocate increased financial resources to improve epidemiological surveillance of the disease, to expand diagnostic capacity, and to advance preventive and therapeutic medical practices among HCW and the community.

## THE ONE HEALTH APPROACH

Because of the complexity of the zoonotic cycle of maintenance in nature and transmission, it is urgent to systematically use the One Health approach as the foundation for each intervention tackling the disease, as there is growing evidence about the benefits of simultaneously addressing the environmental, animal, and human determinants of tick-borne diseases.^23^^,^^35^^–^^37^ Even though many efforts (i.e., binational technical forums and national campaigns to promote awareness about the disease) have been led by federal health authorities to improve the understanding of the multiple factors associated with RMSF, it was underlined that more comprehensive and sustained strategies should be strengthened across the country, particularly in regions of hyperendemicity.

To succeed in the One Health approach, it is necessary to take steps to expand the technical capacity for identifying the geographic patterns of tick species transmitters of rickettsial diseases. Particularly, it has been noted that at least five genera of hard ticks in the Ixodidae family, namely *Amblyomma* spp., *Dermacento*r spp., *Haemaphysalis* spp., *Ixodes* spp., and *Rhipicephalus* spp.,^38^ as well as 17 species of *Rickettsia* have been identified in Mexico, including many that are medically relevant.^39^ Most of these findings are the result of a progressive increase in acarological studies carried out in both the national network of public health laboratories and research centers. This collaboration should be strengthened and expanded, addressing the need to design and implement an acarological and entomological surveillance and monitoring system across the country, serving as a starting point for phenological and other environmental studies to improve interventions for prevention and control of ticks associated with the transmission and of rickettsial pathogens. In this regard, the current presence of *R. rickettsii*, *Rickettsia parkeri*, and *Rickettsia massiliae* along the border region with the United States was noted.^39^^,^^40^ These pathogens are within the spotted fever group of *Rickettsia* (SFGR) and have been found in Mexico in ticks, domestic and wild reservoirs, and humans.^41^^–^^44^

It was also highlighted that the scope of public health research and interventions should be expanded to the urban and sylvatic transmission cycles of RMSF, because regional evidence shows that both cycles can be associated with the occurrence of human and canine cases and clusters.^23^^,^^45^ In this scenario, it was recommended to investigate a major diversity of domestic and wild mammals, mainly dogs, cats, bats, coyotes, opossums, and rabbits, as they have the potential to be infested by hard ticks. Research should include not only small rodents but also medium-sized mammals that are regionally endemic across the country, because little is known at the national level about their role as reservoirs and/or amplifying hosts of different species of *Rickettsia*.^42^^,^^43^^,^^45^^,^^46^

The panelists emphasized the relevance of describing the demographic patterns and mobility of dogs, particularly of free-roaming dogs. This is important because recent scientific evidence has shown that dogs from high-risk areas exhibit higher fecundity and tend to roam more than those from lower-risk areas in a region of endemicity in northwest Mexico.^23^^,^^47^ Overall, free-roaming dogs have higher rates of tick infestation, fertility, and interaction with wild animals. In this sense, the primary goals of programs addressing RMSF should include expanding spay-neuter campaigns of pet dogs and reducing the rate of free-roaming dogs in Mexico to avoid exposure to ticks.^47^^,^^48^ Promoting responsible ownership of pet dogs is a priority to change the epidemiological pattern of RMSF in the country. Additionally, the significance of establishing regulatory mechanisms for the cross-border transportation of animals was discussed. Some alternatives to mitigate the spread of rickettsial pathogens among animals along the U.S.-Mexico border could be the requirement of vaccination records and travel certificates. Recently enacted U.S. regulations now require vaccination and microchipping of dogs, which may help identify infected dogs.^49^ All these facts showed the need to add the veterinarian community as a bridge for education on dog ownership but also to improve the epidemiological surveillance of dogs in the region.

On the other hand, the need to expand the current national list of canine acaricides to improve interventions directed against *Rhipicephalus sanguineus* s.l. and other hard tick species was also emphasized. In this context, there was an extensive discussion of the efficacy and safety of topical acaricides, long-acting tick repellent collars, and systemic acaricides (i.e., isoxazolines). It was recognized that expanding the current list of acaricides would have the potential to protect pet dogs from tick exposure, thereby reducing the transmission of rickettsial pathogens. A call was made to strengthen field investigation of this approach, as well as the allocation of budgetary resources.

The interaction between animals, whether domestic or wild, and people could shape regional differences in epidemiological patterns of RMSF. This should be carefully considered in the design and implementation of medical, animal, and public health programs, systematically incorporating the social determinants of health in each region. Some interventions carried out against the disease in Sonora,^22^ Baja California^50^ and Yucatan^51^ were discussed. These successful experiences, along with others elsewhere,^52^ include integrated interventions based on the following: 1) spraying of safe and effective pesticides, 2) applying tick-repellent collars to dogs, 3) monitoring of the tick community, 4) spay-neuter campaigns for dogs, and 5) education of the community. All of these activities can reduce the magnitude and impact of RMSF, especially when implemented during strategic seasonal periods (early spring to early autumn) with community outreach campaigns and alliances between academia and health authorities.^22^^,^^48^

Finally, the panelists discussed various pathways to better implement the One Health approach to include legislative changes to 1) promote a transdisciplinary vision and multidisciplinary collaborations across different working groups, including empowered communities, and 2) acknowledge that a myriad of biological, environmental, and social determinants exacerbate suffering and fatality, primarily for vulnerable individuals (i.e., children, older adults, and the poor) as well as for impoverished communities.

## RESEARCH ON RMSF

In Mexico, where RMSF is endemic, enhancing the relationship among academia, industry, and government is key for identification of best practices on prevention, diagnosis, and medical care of patients. In this context, it is essential to actively involve health science students in the dissemination of preventive strategies as well as in the knowledge of early symptoms of the disease, which could reduce fatal outcomes.

A relevant discussion took place about the benefits of the promising advances seen in canine and human vaccines for RMSF. For instance, a recent study showed benefits of a whole-cell antigen (WCA) vaccine using *R. rickettsii* inactivated antigens applied to dogs. The dogs that received WCA vaccine did not become infected, whereas those receiving a recombinant vaccine developed disease similar to that of nonvaccinated *R. rickettsii*-infected dogs. Furthermore, the WCA vaccine was also able to reduce the *R. rickettsii* load to nearly undetected levels in several organs and tissues, and it induced bacterial antigen-specific immune responses. Despite these findings, the duration of protection conferred by the WCA vaccine is still unclear.^53^

Similarly, the benefits of an anti-*Rhipicephalus* vaccine based on previous knowledge of the cattle tick *Rhipicephalus microplus* was broadly discussed.^54^^,^^55^ It was also pointed out that the development of a vaccine against *R. sanguineus* s.l. ticks could be commercially attractive, considering the global distribution of this tick and the fact that various other pathogens of veterinary importance, including *Ehrlicha canis* and *Anaplasma platys*, are also transmitted by *R. sanguineus* s.l. Overall, it was noted that potentially effective human^56^ and canine vaccine candidates exist in experimental phases.^53^^,^^57^^–^^59^ However, financial and commercial barriers may limit progress in the development and implementation of these vaccines. To overcome such barriers, it is crucial to test some of those vaccine models in field studies, prioritizing areas with a major burden of RMSF in Mexico. The experts emphasized that effective control of RMSF across the country will not be completely achieved until effective and deployable canine vaccines against *R. rickettsii* and, even more importantly, against the tick *R. sanguineus* s.l. are a reality.

To gain understanding about clinical, epidemiological, and social features in population patterns of RMSF, field investigations should be encouraged within health departments and research centers. In this context, the role of academics, researchers, and clinicians should be expanded beyond diagnostic and medical aspects of cases and outbreaks, both human and animal, and should include innovation in and ascertainment of preventive interventions, such as:

1) Investigating the growing resistance of *R. sanguineus* s.l. to pesticides used at the national level for controlling mosquitoes (i.e., *Anopheles* and *Aedes* genera), which has contributed to suboptimal reduction of tick populations in Mexico.
2) Exploring the efficacy and safety of novel pesticides, both synthetic and natural, aimed at controlling the tick *R. sanguineus* s.l. equally in dogs and the environment.
3) Assessing the efficacy, safety, and equitable access to preventive interventions (i.e., long-acting tick repellent collars, systemic acaricides, wall treatment), especially their scalability and sustainability. Based on a transdisciplinary approach, researchers can prioritize the inclusion of vulnerable individuals and populations.
4) Fostering modeling studies about the impact of environmental changes on the geographical distribution of tick vectors and the reservoirs of the pathogen.
5) Designing validated studies based on the One Health approach targeting high-risk communities across the country, similar to interventions previously carried out in Mexico and the United States.^21^^,^^22^^,^^52^

## CONCLUSION

In Mexico, RMSF must be recognized as an ongoing public health problem, affecting primarily the Mexican states along the U.S. border. Over the past decade in this region, there has been a growing trend in morbidity and an unacceptable high case-fatality rate (30.6%), which demands immediate action to mitigate its deleterious impact, particularly on vulnerable human groups such as children, migrants, older adults, and impoverished communities. In this sense, to maximize the efforts deployed in Mexico against the disease, the experts gathered at the Symposium RMSF 2023 agree that adherence with the following recommendations would contribute positively to 1) understanding of the problem, 2) implementation and evaluation of interventions, and 3) reduction in morbidity and mortality caused by the disease.

## NEXT STEPS

**Figure 2.**
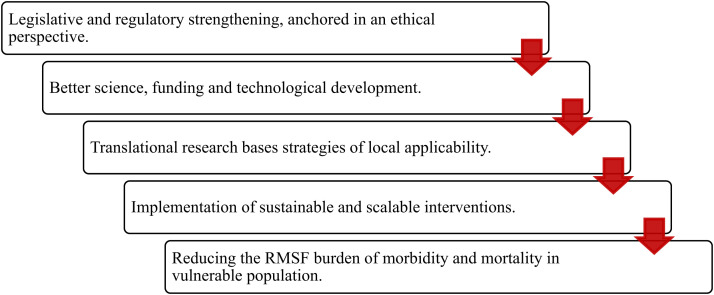
A sequence of essentials for the prevention and control of RMSF in Mexico. RMSF = Rocky Mountain spotted fever.

1. Improving the epidemiological surveillance system at both the regulatory and operative levels. This goal requires strengthening knowledge and skills of physicians to suspect, treat, confirm, and notify cases and outbreaks. Furthermore, there is an urgent need for systematic evaluation of the quality of statistics to improve epidemiological estimations about RMSF and other SFGR. Additionally, it is necessary to incorporate the surveillance of social determinants of health, as they play a crucial role in the causal pathway of the disease.
2. Despite the recent advances witnessed across the country, there is a need to enhance diagnostic capacity to confirm cases and outbreaks of RMSF. The search for *R. rickettsii* should include ticks, animals, and humans, requiring the implementation of novel techniques, training of health personnel, and strengthening the infrastructure of state and regional laboratories. Improving the training of health personnel who carry out prevention, diagnosis, and medical care actions in regions of endemicity is a fundamental strategy. This training should include doctors from public and private institutions, particularly those who care for patients in offices attached to drug stores, where 19.9% of the population is served.^60^
3. In this context, innovative diagnostic methods such as the lateral-flow assay, the loop-mediated isothermal amplification PCR, and other techniques from molecular biology should be explored and implemented to guide clinical and public health interventions.^32^^,^^61^^,^^62^
4. Doxycycline remains the antibiotic of choice for treating patients in all age groups with RMSF, and its efficacy is proven when administered in a timely manner.^31^^,^^63^ Improved access to intravenous and pediatric formulations of this antibiotic could dramatically reduce morbidity and mortality of patients with RMSF. Urgent implementation of technical and regulatory mechanisms at the national level are necessary to acquire and distribute adequate quantities of these formulations of doxycycline, especially in regions of hyperendemicity, for the medical care of the youngest and the most severely ill patients.
5. The complexity of the zoonotic transmission chain of RMSF requires the expansion of educational strategies targeting health personnel, veterinary services, and the community. Experts emphasized the need to increase knowledge about *Rickettsia*-host-tick interactions to implement effective actions against RMSF and other SFGR. It was unanimously agreed that the One Health approach is the best alternative to deal with the several determinants of rickettsioses and that it can guide clinical and sanitary interventions.^64^ A transdisciplinary vision and a multidisciplinary action based on governmental, academic, and societal alliances are urgently required.
6. For a more comprehensive understanding of epidemiological patterns of RMSF, it is critical to further study interactions between ticks and hosts. To make this feasible, it is necessary to design and operate an acarological surveillance and monitoring system across the country. This system would produce information to anticipate the occurrence of regional foci of rickettsial agents and other tick-borne pathogens.
7. Actions and strategies from health promotion should be anticipatory, sustainable, and culturally appropriate, mainly those targeted to vulnerable populations in rural and urban communities. Some key aspects to consider include: a) increasing awareness about risks related to tick exposure, b) providing human and canine protection against tick infestation, c) promoting responsible ownership of pet dogs, d) training of both health personnel and community regarding early symptoms of the disease, and e) emphasizing the importance of seeking medical attention promptly.
8. There is a need to improve health care of pet dogs mainly in those localities with hyperendemic foci, particularly through sustainable spay/neutering campaigns, interventions to reduce their exposure to ticks, and raising awareness among owners to keep dogs on their property.
9. The participation of the scientific community is pivotal to improve: a) understanding the determinants and outcomes of the disease, b) validating novel diagnostic tools as well as the efficacy of supportive therapies for severe cases, c) advancing the development of innovative and safe acaricides, and d) developing and evaluating canine vaccines against *Rickettsiae* and ticks.
10. The current situation of RMSF in Mexico, particularly in the states bordering the United States, must be addressed as a binational public health problem. Continued collaboration between U.S. and Mexican health officials and scientists will enhance and synergize opportunities to reduce the burden of disease occurring in that region.^64^^–^^66^
11. Finally, the panelists discussed various pathways to better implement the One Health approach to include: a) promoting a transdisciplinary vision and multidisciplinary collaborations across different working groups, including empowered communities; and b) acknowledging that a myriad of biological, environmental, and social determinants exacerbate suffering and fatality, primarily for vulnerable individuals (i.e., children, older adults, and the poor) as well as for impoverished communities (Figure 2).
